# Ovarian hormone fluctuations across the menstrual cycle, effects on brain structure and function, and affective psychopathology

**DOI:** 10.1192/j.eurpsy.2026.10328

**Published:** 2026-07-31

**Authors:** B. Pletzer

**Affiliations:** University of Salzburg, Salzburg, Austria

## Abstract

**Abstract:**

The menstrual cycle represents a unique opportunity to study the effects of ovarian hormones on the brain given the distinct peaks of estradiol and progesterone in the ovulatory and luteal cycle phase. Evidence of neuroplastic effects of estradiol at the cellular level is reflected in hippocampal volume changes during the ovulatory phase, while progesterone increases during the luteal phase relate to changes in basal ganglia volumes. Likewise fronto-striatal activity increases with progesterone during the luteal phase across a variety of tasks and imaging modalities. Nevertheless these changes in brain structure and activity remain very modest in effect size. More impressive however are changes in brain connectivity, which have been replicated across a variety of methods and modalities. While cognitive performance remains relatively stable along the menstrual cycle, neural networks adapt to changing hormonal milieaus, shaping new pathways for information through the brain. These changes may even be strong enough to predict a woman’s cycle phase from her brain connectivity patterns. However, mental health trajectories along the menstrual cycle are highly individualized reflected in premenstrual symptomatologies. The talk will characterize the female brain across different menstrual cycle phases and summarize behavioral evidence related to premenstrual symptom trajectories along the menstrual cycle. Finally it will discuss a road forward to linking the two phenomena.

**Disclosure of Interest:**

None Declared

